# A cyclic nano-reactor achieving enhanced photodynamic tumor therapy by reversing multiple resistances

**DOI:** 10.1186/s12951-021-00893-6

**Published:** 2021-05-21

**Authors:** Peng Liu, Yanbin Zhou, Xinyi Shi, Yu Yuan, Ying Peng, Surong Hua, Qiange Luo, Jinsong Ding, Yong Li, Wenhu Zhou

**Affiliations:** 1 Xiangya School of Pharmaceutical Sciences, Central South University, Changsha, 410013 Hunan China; 2 Department of Pediatric Surgery, Hunan Childrens Hospital, Changsha, 410004 Hunan China; 3 Department of General Surgery, Peking Union Medical College Hospital, Beijing, 100730 China

**Keywords:** Nanomedicine, Targeting, Tumor hypoxia, Drug resistance, Starvation therapy, GSH depletion, Oxygenation, Metalorganic-frameworks

## Abstract

**Background:**

Photodynamic therapy (PDT) is a clinically implemented modality to combat malignant tumor, while its efficacy is largely limited by several resistance factors from tumor microenvironment (TME), such as hypoxia, anti-oxidant systems, and ATP-dependent tumor adaptive resistances. The aim of this work is to construct a multifunctional nanoplatform to remodel multiple resistant TME for enhanced PDT.

**Results:**

Here, a targeting nano-reactor was facilely constructed to reverse the multiple resistances of PDT by incorporating glucose oxidase (GOx) and chlorin e6 (Ce6) into poly (D, L-lactic-co-glycolic acid) (PLGA)/ metalorganic framework (MOF) coreshell nanoassembly, with surface deposition of hyaluronic acid (HA) stabilized MnO_2_. The nano-reactor could selectively target tumor cells by virtue of surface HA modification, and once internalization, a few reactions were initiated to modulate TME. Glucose was consumed by GOx to inhibit ATP generation, and the produced H_2_O_2_ was catalyzed by MnO_2_ to generate O_2_ for tumor hypoxia alleviation and photodynamic sensitization, and glutathione (GSH) was also effectively depleted by MnO_2_ to suppress the tumor antioxidant defense. Consequently, the nano-reactor achieved robust PDT with amplified tumor therapy via intravenous injection.

**Conclusions:**

This nano-reactor offers a multifunctional nanoplatform to sensitize TME-limited tumor treatment means via reversing multiple resistances.

**Grpahic abstract:**

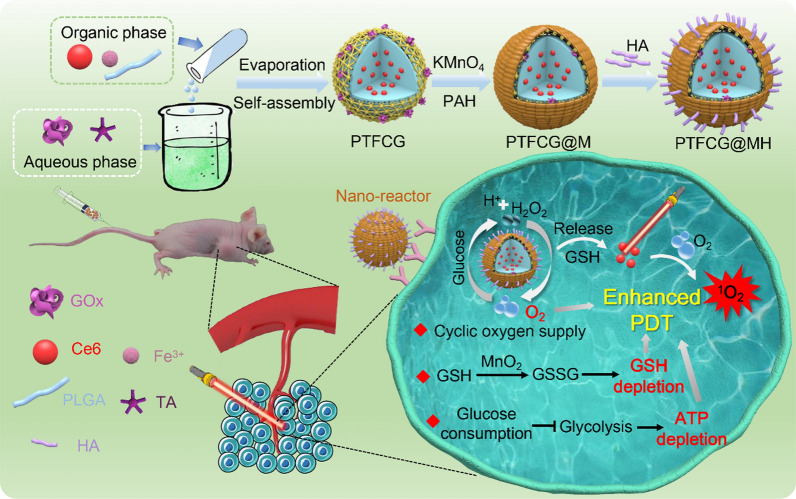

**Supplementary Information:**

The online version contains supplementary material available at 10.1186/s12951-021-00893-6.

## Background

Photodynamic therapy (PDT) has gain tremendous fundamental and translational attention for tumor therapy, owing to its advantages of low systemic toxicity, non-invasive, spatial and temporal controllable activation [1, 2]. During the process of PDT, the photosensitizers (PSs) are activated by light to convert oxygen into toxic reactive oxygen species (ROS), which bind and dis-functionalize some bio-macromolecules in tumor cells, such as DNA and lipid, resulting in cell apoptosis or necrosis, immune responses and microvascular damage [3,7]. Such process is highly efficient to kill cancer at cellular level, and some PSs, such as verteporfin, 5-ALA and temoporfin, have been demonstrated as promising candidate for clinical translation [8, 9]. However, while PDT has been accepted for several types of cancer in clinic [10], its widespread implementation is still hindered by various biological limitations. Aside from the inherent barrier of light penetration that can be partially addressed by deep PDT techniques, tumor resistances are the main mechanism to weaken the efficacy of PDT [11].

Tumor cells can resist PDT by different pathways due to the complexity of tumor microenvironment (TME), in which the most well-known feature is hypoxia. Hypoxic cells are~threefold more resistant to ROS damage than aerobic cells [12], and more importantly, hypoxic environment could directly decrease the PDT efficacy by blocking the oxygen supply [13, 14]. Whats more, the PDT process would further aggravate tumor hypoxia through oxygen consumption and vascular damage, which activates hypoxia inducible factor-1 (HIF-1) survival pathway, thus causing PDT resistance [15, 16]. At the same time, cancer cells are equipped with antioxidant defense systems, in which the most abundant one is glutathione (GSH), to scavenge the ROS and thus counteract ROS-mediated injury [17,19]. In addition, tumor cells could generate adaptive resistance toward PDT through upregulation of drug efflux proteins, heat shock proteins (HSPs), and DNA repair proteins [20,22]. It has been reported that numerous drug efflux proteins, such as P-glycoprotein (P-gp), and ATP-binding cassette super-family G member 2 (ABCG2), have been implicated in PDT resistance via pumping out PSs before their action [23].

To reverse PDT resistance, various nano-systems capable of modulating TME have been developed. For example, replenishment of oxygen is a commonly employed strategy to alleviate hypoxia, which can be achieved by either oxygen delivery (using hemoglobin or perfluorocarbons) or endogenous oxygen generation (through catalytic converting tumor abundant H_2_O_2_ into O_2_) [24,26]. Oxygen generation can be further boosted by cascade equipping the nano-system with glucose oxidase (GOx) to supplement H_2_O_2_ substrate [27]. To augment PDT efficacy, several nano-vehicles were also designed to suppress the tumor antioxidant defense and restore the ROS damage effect of PDT via antioxidants depletion [28, 29]. Moreover, inhibition of ATP was reported to sensitize PDT by inhibiting drug efflux and aggravating PDT-induced DNA damage [30, 31]. Ideally, simultaneous remodeling various TME to alleviate multiple biological resistances is preferred for optimized PDT, considering the cunning nature of cancer. Unfortunately, the development of nanoparticles with multifunctionalities often requires sophisticated materials design and complicated preparation procedure, thus imposing the difficult of cost-effective, reproducible, and scalable production.

**Scheme 1:**
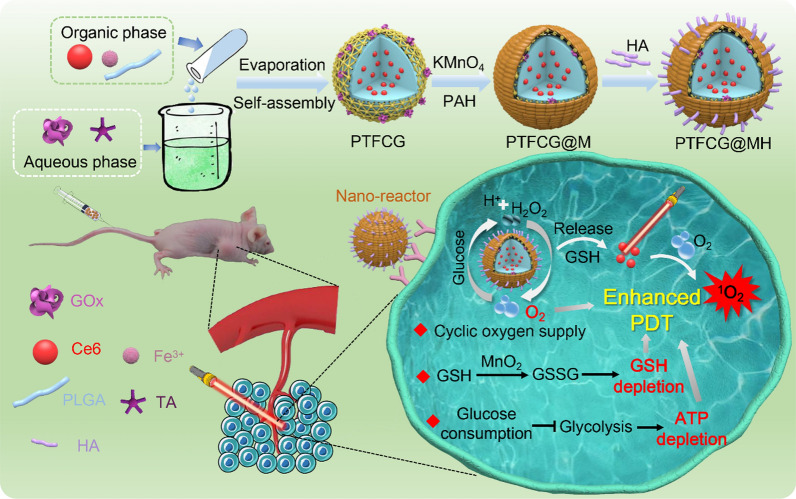
Schematic illustration the preparation of core-sell nano-reactor, and its targeted delivery in vivo for enhanced PDT against tumor by reversing multiple resistances

To tackle this, we designed and facilely prepared a coreshell nano-reactor for enhanced PDT via simultaneous oxygenation, antioxidant suppression, and ATP depletion (Scheme 1). Tannic acid (TA), ferric iron (Fe^3+^) and poly (D, L-lactic-co-glycolic acid) (PLGA) were assembled into coreshell structure via hydrophobic, - staking, and electrostatic interactions [32,34], in which chlorin e6 (Ce6, a widely used PS) and GOx were co-loaded. MnO_2_ was deposited on particle surface and stabilized by hyaluronic acid (HA) through layer-by-layer electrostatic adsorption. In our system, the MnO_2_ played dual roles of self-oxygen supply and GSH depletion via its catalase-mimic activity and oxidbillity, respectively. The GOx boosted the oxygenation by in-situ generation of H_2_O_2_, and its capability of glucose consumption blocked the energy supply to decrease ATP generation. All these functions collectively remodeled the TME from different aspect to sensitize PDT efficacy, which has been demonstrated by solution, in vitro and in vivo experimental results. The nanosystem could passively accumulate into tumor, actively internalize tumor cells via HA-mediated targeting, and impose potent PDT effect via reversing multiple resistances.

## Materials and methods

### Materials

Chlorin e6 (Ce6) and Nile red (NR) were obtained from Frontier Scientific, Inc. (Utah, USA). PLGA (Mw: 20kDa) was purchased from Daigang Biomaterial Co., Ltd. (Jinan, China). Hyaluronic acid (HA, 7kDa) was purchased from Lifecore Biomedical Company (MN, USA). Poly (allylamine hydrochloride) (PAH), tannic acid (TA), glucose oxidase (GOx), singlet oxygen sensor green (SOSG), and 3-(4, 5-dimethylthiazol-2-yl)-2, 5-diphenyl tetrazolium bromide (MTT) were provided by Sigma-Aldrich (Saint Louis, MO, USA). Live & dead viability/cytotoxicity assay kit was obtained by Invitrogen (NY, USA). Ellmans reagent and 4, 6-diamidino-2-phenylindole (DAPI) were provided by Solarbio Biotech, Co., Ltd. (Beijing, China). Ferric chloride hexahydrate (FeCl_3_6H_2_O), N-acetylcysteine (NAC) and 2 7'-dichlorofluorescin diacetate (DCFDA) were obtained from Sinopharm Chemical Reagent Co., Ltd (Shanghai, China). DMEM medium, fetal bovine serum, streptomycin/penicillin and TrypsinEDTA were provided by GIBCO (NY, USA). The 3,5-dinitrosalicylic acid (DNS) reagent was purchased from Coolaber Biotech, Co., Ltd. (Beijing, China). ROS-ID hypoxia detection kit was provided by Enzo Life Sciences Inc. (NY, USA).

### Preparation of PTFCG@MH

Ce6, FeCl_3_ and PLGA were mixed in acetone at a concentration of 400g/mL, 400g/mL and 2mg/mL, respectively. Then, 1mL of such mixture was added dropwise to 5mL aqueous solution containing 320g/mL TA and 20g/mL GOx under constant sonication. The solution was stirred at 30C for 3h to evaporate acetone. The PTFCG was separated by centrifugation (16,000rpm, 25min) and washed by deionized water. To prepare PTFCG@MH, 50 L PAH (20mg/mL) was mixed with 1mL PTFCG solution and mild stirred for 30min. Then, 25 L potassium permanganate (10mg/mL) was added and stirred for another 1h. After centrifugation (16,000rpm for 25min) and washing with deionized water, the PTFCG@M was obtained. Finally, 500 L HA (20mg/mL) was introduced into 1mL of PTFCG@M solution and stirred for 1h. Excess HA was removed by centrifugation (16,000rpm, 25min), and PTFCG@MH was collected.

### Characterizations of PTFCG@MH

The particle sizes and potential of PTFCG, PTFCG@M and PTFCG@MH were measured by a Malvern Zeta Sizer Nano series (Malvern, UK) at 25C. The morphologies and element content were investigated by TEM-EDS (Titan G2 60300, FEI, USA). For TEM-EDS analysis, the PTFCG or PTFCG@MH solution was dropped onto carbon film-coated copper grid and observed under TEM-EDS instrument. The UVvis absorption spectra were carried out on UVvisible spectrophotometer (Shimadzu, Japan). The fluorescence spectra were collected using FL-2700 spectrofluorometer (HITACHIH). The Ce6 concentration was determined by the UVvisible spectra spectrophotometer at 640nm. The loading amount of GOx was estimated using bicinchoninic acid (BCA) protein assay (Beyotime) and the protocol was provided by the supplier. The drug loading (DL) of Ce6 and GOx in the PTFCG@MH was calculated as follows:

$${\text{DL}}\, = \,{\text{amount of loaded drug in the PTFCG}}@{\text{MH }}/{\text{ weight of PTFCG}}@{\text{MH }}(\mu {\text{g}}/{\text{mg}}).$$

### In vitro release of Ce6

The PTFCG@MH solution ([Ce6]=200g/mL) was dispersed in 5mL of various dissolution media (10mM PBS buffer, 10mM PBS buffer containing 10mM GSH). The mixtures were shaken gently in a shaking incubator at 37C. At predetermined time intervals (0, 1, 2, 4, 8, 12h), 0.5mL of samples were withdrawn and centrifuged (16,000rpm, 25min). The supernatant was collected, and the amount of Ce6 was quantified by UV absorbance quantification (=640nm).

### Cyclic reaction of PTFCG@MH

The catalytic activity of GOx was determined by measuring the glucose consumption and pH change in presence of glucose. For the glucose consumption, the PTF@MH or PTFCG@MH solution ([GOx]=4g/mL) was mixed with glucose (10mM) in the presence or absence of H_2_O_2_ (100M) at 37C, then the glucose concentration was detected by DNS reagent (Coolaber) within 1h and the protocol was provided by the supplier. For the pH change, the glucose (10mM) was added into PTFCG or PTFCG@MH solution ([GOx]=4g/mL) at 37C, then the pH value was measured by a pH meter (PHSJ-4F, INESA, China) within 30min. To monitor the oxygen generation under different acidic conditions, PTFCG@MH ([GOx]=4g/mL) was dispersed in 10mM PBS buffer (pH 5.0, 6.0, and 7.0) containing 1mM H_2_O_2_. At predetermined time points, the dissolved O_2_ was detected using the portable dissolved oxygen meter (JPBJ-609L, INESA, China). Moreover, the PTFCG or PTFCG@MH ([GOx]=4g/mL) was mixed with glucose (10mM) in the presence or absence of H_2_O_2_ (100M) and laser irradiation (ADR-1805 Laser, 100 mW/cm^2^, 635nm), then the dissolved O_2_ was measured within 160s.

### The ^1^O_2_ generation analysis

PTF@MH or PTFCG@MH ([Ce6]=1g/mL) was added into SOSG solution (2.5M), followed by adding H_2_O_2_ (10mM). Then, a continuous laser (100 mW/cm^2^, 635nm) was performed at predetermined time points (0, 1, 2, 4, 6, 8, 10min), and fluorescence intensity was detected immediately by fluorescence spectroscopy (Ex=490nm, Em=525nm).

### Cell culture

MDA-MB-231 and HUVEC cells were cultured under a humidified atmosphere of 5% CO_2_ at 37C using DMEM and RPMI 1640 complete medium, respectively. The complete medium contains 10% FBS (GIBCO, USA), 1% penicillin/streptomycin (100 U/mL, Solarbio Bioteh).

### Cellular uptake

The NR-loaded nanoparticles were prepared following the method described above by replacing Ce6 with NR. The cells were seeded in Petri dish (35mm) at 510^4^ cells/cm^2^ and cultured overnight. With or without pre-treatment of free HA (10mg/mL) for 1h, the cells were incubated with NR-loaded nanoparticles or free NR ([NR]=2g/mL) for another 2h. Next, the cells were washed with pre-cooled PBS and fixed with 4% paraformaldehyde. After staining by DAPI (1g/mL), the cells were imaged under confocal microscope (LSM780 NLO, Zeiss, Germany).

To study the endocytosis mechanism, the cells were seeded in 6-well culture plate (210^5^ cells/well). After pretreating with chlorpromazine (10g/mL), nystatin (15g/mL) or colchicine (5g/mL) for 1h, the cells were incubated with NR-loaded nanoparticles ([NR]=2g/mL) for 2h. Then, the cells were washed with pre-cooled PBS, digested by TrypsinEDTA and analyzed using flow cytometry (FACSVerse, BD, USA).

### Intracellular O_2_ consumption and ROS detection

ROS-ID and DCFDA were used to detect the intracellular O_2_ consumption and ROS generation inside cells. The MDA-MB-231 cells were seeded in 24-well culture plate (10^5^ cells/well) and cultured overnight. After sealing by liquid paraffin, the cells were incubated with PTFCG@MH, PTF@MH, PTFCG, or free Ce6 ([Ce6]=1g/mL) for 2h. Then, laser irradiation (635nm, 100 mW/cm^2^) was applied for 2min. With PBS washing for three times, the mixture of ROS-ID (0.5M) and DCFDA (10M) was added into each group for 30min further incubation. After washing with pre-cooled PBS, fluorescence inside cells was observed by fluorescence imaging system (NIKON, Ti-S, Japan).

### Intracellular ATP and GSH detection

MDA-MB-231 cells were seeded in 12-well plate at a density of 210^5^ cells per well for 12h incubation. Then, various formulations with different concentrations were added for 24h incubation. After that, the cells were collected and lysed using ATP lysis buffer, and ATP concentration was measured by ATP assay kit (Beyotime). Likewise, the cells were collected and lysed using the Triton-X 100 cell lysis buffer, and GSH concentration was detected by the Ellmans reagent (Solarbio). The numbers of cells were standardized by measuring total protein concentration using BCA protein assay (Beyotime).

### In vitro cytotoxicity studies

MDA-MB-231 cells were seeded in a 96-well plate (5000 cells/well) for overnight incubation. The cells were treated with PTFCG or PTFCG@MH at different concentrations for 24h, followed by laser irradiation for 1min (100 mW/cm^2^, 635nm). After 24h, MTT solution (1mg/mL) was added for another 4h incubation. The medium was replaced with 100 L of DMSO. The absorbance values at 570nm were recorded to calculate the cell viability. Then, the Live/Dead assay was carried out by calcein AM/propidium iodide double staining. The cells were seeded and treated as described above. After laser irradiation (100 mW/cm^2^, 635nm), the fresh medium containing calcein AM (2M) and propidium iodide (8M) was added for 20min incubation. Finally, the cells were imaged by fluorescence imaging system (NIKON, Ti-S, Japan).

### In vivo/ex vivo fluorescence imaging

Healthy female Balb/c mice (aged 46weeks) were purchased from the Laboratory Animal Center of Central South University. All experimental procedures were carried out in accordance with the Regulations for the Administration of Affairs Concerning Experimental Animals of China, and approved by the Ethics Committee for Research in Animal Subjects at Xiangya School of Pharmaceutical Sciences of Central South University. MDA-MB-231 tumor-bearing mice were obtained by subcutaneously injecting a cells suspension in PBS (10^6^ cells) into the right armpit of mice. When the tumor volume reached~100mm^3^, the mice were treated with different formulations.

For in vivo/ex vivo imaging, the tumor-bearing mice were intravenously injected with free Ce6 or PTFCG@MH (100 L, [Ce6]=2.5mg/kg), and the fluorescence images were taken by an optical imaging system (IVIS Lumina, PerkinElmer, USA) at 1h and 24h post-injection. The mice were sacrificed at 24h after injection, and the major organs were collected for ex vivo imaging. The images were analyzed using Living Imaging Software (IVIS Lumina LT, PerkinElmer, USA).

### In vivo antitumor study

The tumor-bearing mice were randomly divided into 5 groups and injected intravenously with (1) PBS, (2) Ce6+Laser, (3) PTFCG+Laser, (4) PTFCG@MH, (5) PTFCG@MH+Laser (100L, [Ce6]=2.5mg/kg) at day 0 and 4. The irradiation groups were exposed to laser (100 mW/cm^2^, 635nm) for 5min at 24h after injection. The tumor volume was recorded and calculated as follows: V=(lengthwidth^2^)/2. In addition, the body weights were also obtained. At day 14, all the mice were sacrificed, and the tumors were collected and weighed. For histology analysis, the major organs and tumors were extracted and immersed in 4% formaldehyde, embedded in paraffin, sectioned, stained with H&E and observed by an optical microscope (Leica, German).

### Immunofluorescence analysis

The expression of HIF-1 protein was investigated by immunofluorescence assay. Nude mice bearing MDA-MB-231 tumors were sacrificed at 48h after various treatments mentioned above. Tumor tissues were collected for preparing slices of tumor frozen sections. Then, the slices were stained with HIF-1 antibody (mouse polyclonal to HIF-1, Abcam) at 4C overnight, followed by adding the secondary antibodies conjugated with FITC for 1h incubation. After cell nuclei staining with Hoechst 33,258 for 20min, the tumor sections were imaged by confocal fluorescence microscope (LSM780 NLO, Zeiss, Germany).

### Statistical analysis

Data were presented as meanSD. Analysis was performed using Graphpad Prism 5 software. One-way ANOVA analysis of variance was conducted to determine the statistical significance of various groups. *P* values<0.05 was regarded as statistically significant.

## Results and discussion

### Nanoparticles preparation and characterization

**Fig. 1:**
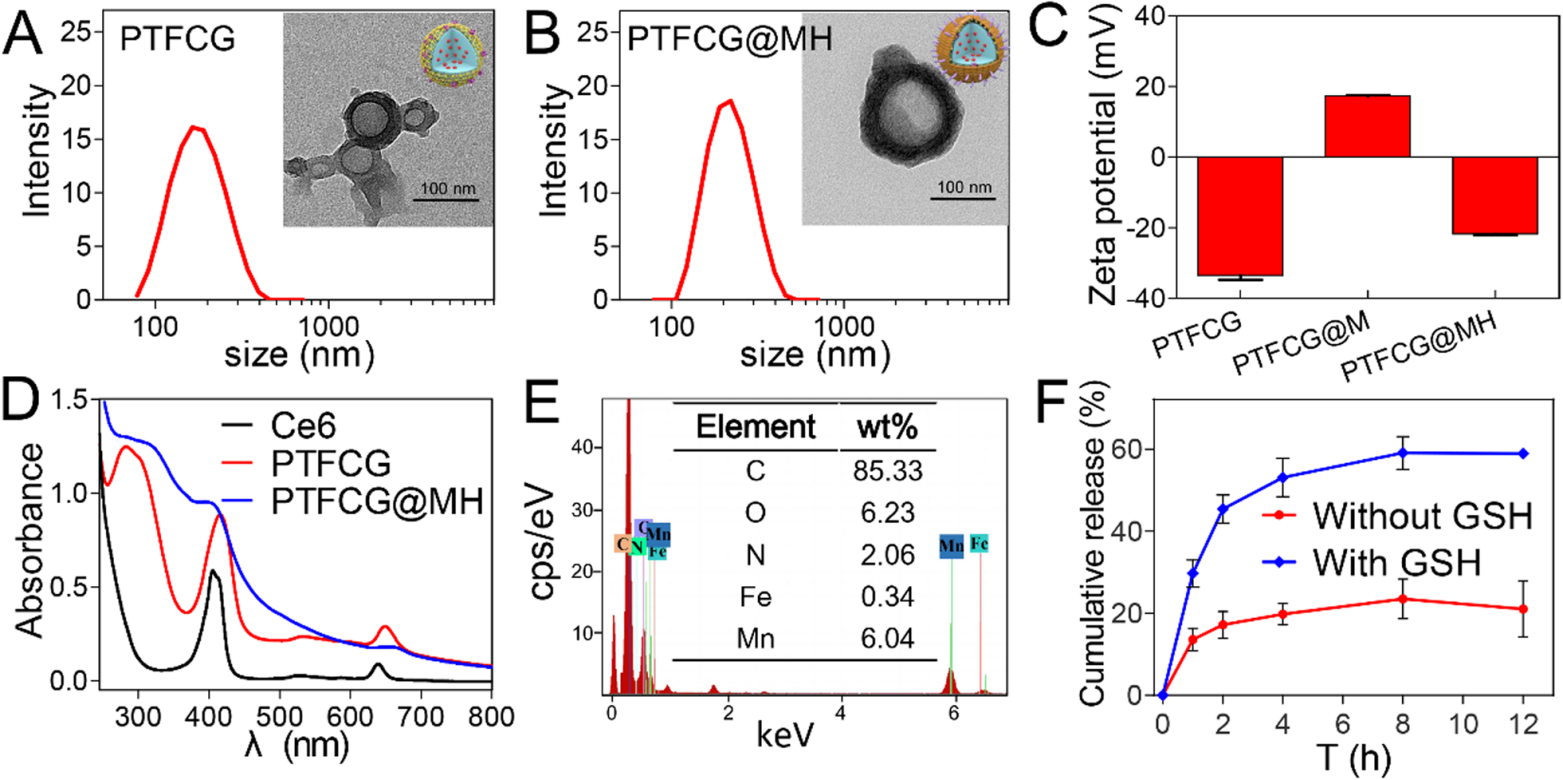
Dynamic sizes and TEM images of **A** PTFCG and **B** PTFCG@MH. **C** The potential of PTFCG, PTFCG@M and PTFCG@MH. **D** UVvis absorbance spectra of Ce6, PTFCG and PTFCG@MH. **E** Energy dispersive X-Ray spectroscopy (EDS) analysis of PTFCG@MH. **F** In vitro Ce6 release from PTFCG@MH with or without addition of GSH (10mM)

The GOx and Ce6 were co-loaded into nanocomposites (PTFCG) using a solvent exchange and evaporation method (Scheme 1), in which the ethanol solution (containing Ce6, PLGA and FeCl_3_) was dropwise added into the aqueous phase (with TA and GOx). During organic evaporation, the TA-Fe metalorganic frameworks (MOFs) were coated on the surface of hydrophobic PLGA nano-core for particle stabilization, and the resulting PTFCG displayed a dynamic diameter of~175nm (Fig.1A, Additional file 1: Figure S1) with potential of 33.4mV (Fig.1C). From the TEM image, the PTFCG displayed a roughly spherical morphology with an obvious coreshell structure (inset in Fig.1A). To further deposit MnO_2_ on particle surface, a PAH solution was added, followed by adding KMnO_4_, allowing for in-situ growth of MnO_2_ on nanoparticle surface to yield PTFCG@M. Compared with PTFCG, the potential of PTFCG@M was reversed to positive (+17.3mV) (Fig.1C), which allowed for the subsequent HA coating via electrostatic attraction (termed PTFCG@MH). The successful HA modification was evidenced by the decrease of particle charge to negative (21.7mV) (Fig.1C), which is beneficial for enhanced colloidal stability. [35] The resulting PTFCG@MH nanoparticles displayed a dynamic diameter of~205nm (Fig.1B), while the size measured by TEM was~160nm (inset in Fig.1B, Additional file 1: Figure S2). It is reasonable as DLS measurement would distort the particle size being observed due to the particle surface hydration. Importantly, the HA modification not only significantly increased the colloidal stability (Additional file 1: Figure S3), [36] but also rendered the nanoparticles with tumor targetability (vide infra).

According to the UVvis absorbance spectra, PTFCG had characteristic Ce6 peaks at 404nm and 640nm, indicating the successful Ce6 loading (Fig.1D). For PTFCG@MH, a wide absorbance band in the range from 250400nm appeared, which was ascribed to the MnO_2_. To confirm the MnO_2_ coating, the energy dispersive X-Ray spectroscopy (EDS) was conducted, and a high Mn element content (~6%) was observed (Fig.1E). The drug loading (DL) for Ce6 and GOx was measured to be 108.4g and 24.6g per mg of PTFCG@MH, respectively. Interestingly, after encapsulation into nanoparticles, the intrinsic fluorescence of Ce6 was significantly quenched, especially for PTFCG@MH (Additional file 1: Figure S4). However, such quenched fluorescence can be largely recovered upon addition of GSH, indicating GSH-responsive release of Ce6. To confirm this, Ce6 release behavior from PTFCG@MH was investigated (Fig.1F). In absence of GSH, the Ce6 showed~20% accumulative release, while a burst drug release was observed after adding GSH, with~60% Ce6 release within 8h. Therefore, such nanosystem showed a triggered activation of photodynamic activity in response to intracellular stimulus, thus minimizing the potential phototoxicity of Ce6 during in vivo delivery.

### A cyclic reaction nano-reactor

**Fig. 2:**
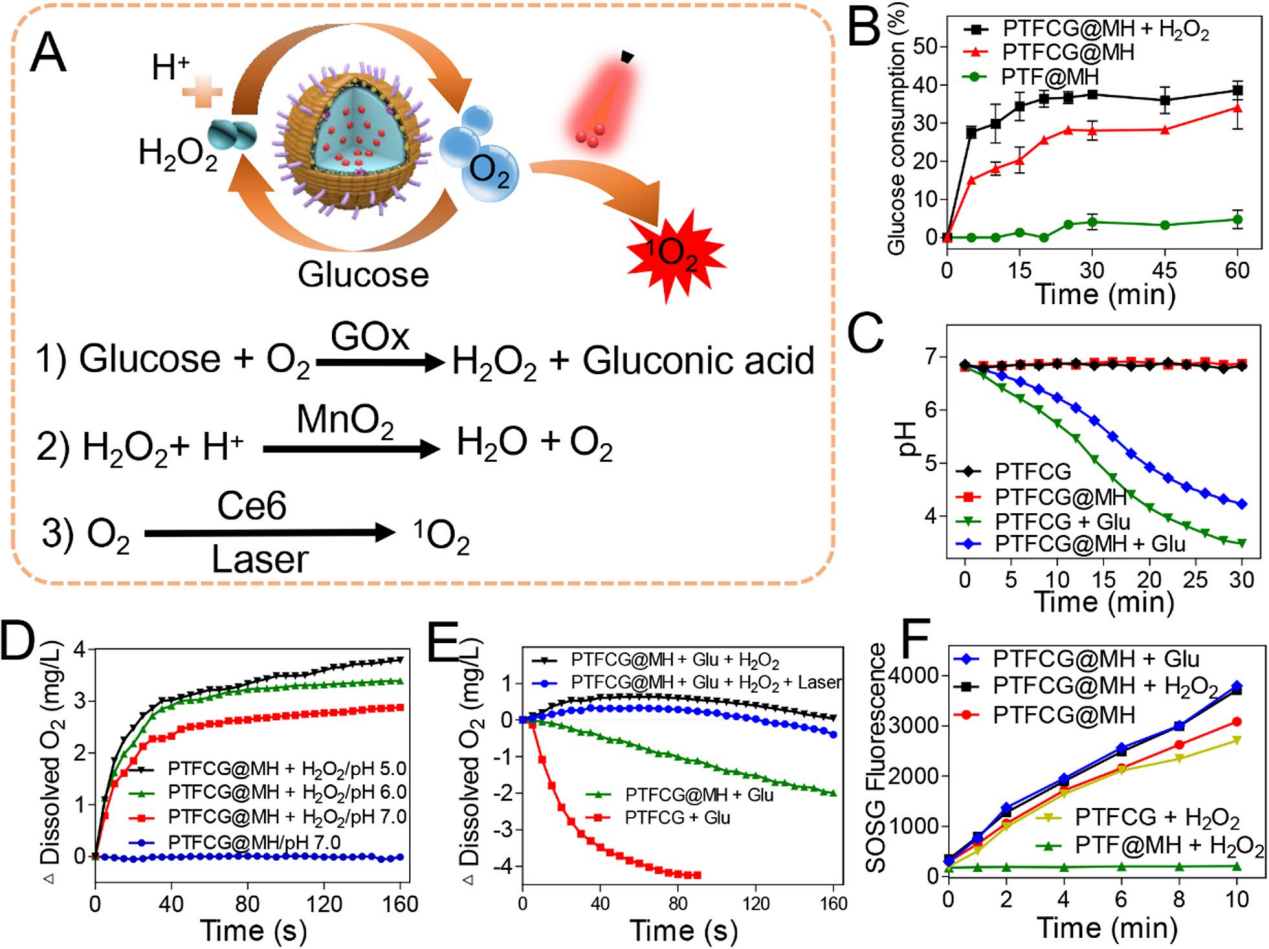
**A** Schematic illustration of cyclic reaction of PTFCG@MH. **B** Glucose consumption kinetics catalyzed by different formulations and conditions. **C** The kinetics of pH change for PTFCG@MH and PTFCG in absence or presence of glucose. **D** The kinetics of oxygen generation for PTFCG@MH in absence or presence of H_2_O_2_ at different pH values. **E** Kinetics of dissolved oxygen variation for PTFCG@MH and PTFCG under different conditions. **F** The ^1^O_2_ generation kinetics for PTFCG@MH, PTFCG and PTF@MH in the presence or absence of H_2_O_2_ or glucose under laser irradiation. The concentration of glucose and H_2_O_2_ was 10mM and 100M, respectively

As illustrated in Fig.2A, our nanosystem was designed with a few cyclic reactions to enhance the Ce6-based PDT. GOx consumes glucose (Glu) to block the energy (ATP) supply, and the concomitantly produced H_2_O_2_ is decomposed by the catalase-mimic MnO_2_ to generate O_2_, which in turn boosts the ^1^O_2_ production under laser illumination. To demonstrate this concept, each reaction was tested individually.

The catalytic activity of GOx was first measured by monitoring the glucose consumption (Fig.2B). After 60min incubation, PTFCG@MH decreased the glucose level by 34%, while the nanoparticles without GOx loading (termed PTF@MH) did not show any glucose consumption. Therefore, GOx maintained its catalytic activity after being loaded into nanoparticles. Moreover, the glucose depletion of PTFCG@MH was further strengthened after adding H_2_O_2_, which can be ascribed to the decomposition of H_2_O_2_ by PTFCG@MH to supply O_2_. MichaelisMenten constant (K_M_) was calculated to quantify the reaction kinetics, and the K_M_ value of PTFCG@MH+H_2_O_2_ and PTFCG@MH turned out to be 6.35 and 10.02mM (Additional file 1: Figure S5), indicating the promoted glucose consumption by H_2_O_2_. The activity of GOx can also be monitored by the pH decrease due to the generation of gluconic acid byproduct. Upon addition of glucose, both PTFCG and PTFCG@MH showed a gradual pH decrease over time (Fig.2C). Notably, PTFCG@MH exhibited a relative lower pH decrease rate than that of PTFCG. This is likely due to that the surface deposited MnO_2_ impeded accessibility of glucose into GOx-loaded nanocore. In addition, the MnO_2_ could neutralize H^+^ to slow down pH decrease rate.

Next, the catalase-mimic activity of MnO_2_ was tested by monitoring O_2_ production in presence of H_2_O_2_ (Fig.2D). As a control, the PTFCG@MH alone did not show any O_2_ generation. However, a rapid increase of the dissolved O_2_ was observed upon addition of H_2_O_2_. We also studied the effect of pH, and a marginal increase of catalytic rate was seen with the decrease of pH from 7.0 to 5.0. Overall, the catalytic efficiency was relatively higher under acidic tumor microenvironment than physiological conditions.

After confirming the single catalytic reaction of GOx and MnO_2_, we next explored the catalytic circulation by measuring the oxygen variation (Fig.2E). For PTFCG, the dissolved O_2_ was quickly decreased within 100s upon addition of glucose. For the PTFCG@MH, by contrast, the O_2_ consumption rate significantly lessened due to the self-oxygen generation activity of MnO_2_, demonstrating the cyclic oxygen supply. Moreover, the O_2_ balance was achieved upon further addition of 100M H_2_O_2_ (which mimics the tumor microenvironment) [37], and such O_2_ generation efficiency is strong enough to support the PDT (Fig.2E, blue trace).

With cyclic oxygen supply, we next explored the enhanced PDT effect by measuring the single oxygen (^1^O_2_) generation using singlet oxygen sensor green (SOSG) as a fluorescent indicator (Fig.2F). In presence of H_2_O_2_, the production of ^1^O_2_ was obviously increased for PTFCG@MH, and the addition of glucose could also strengthen the PDT effect. As a control, the PTFCG group showed lower ^1^O_2_ generation due to the lack of oxygen supply. All these results demonstrated the capability of cyclic nano-reactor for boosting PDT effect of Ce6.

### Cellular uptake

**Fig. 3:**
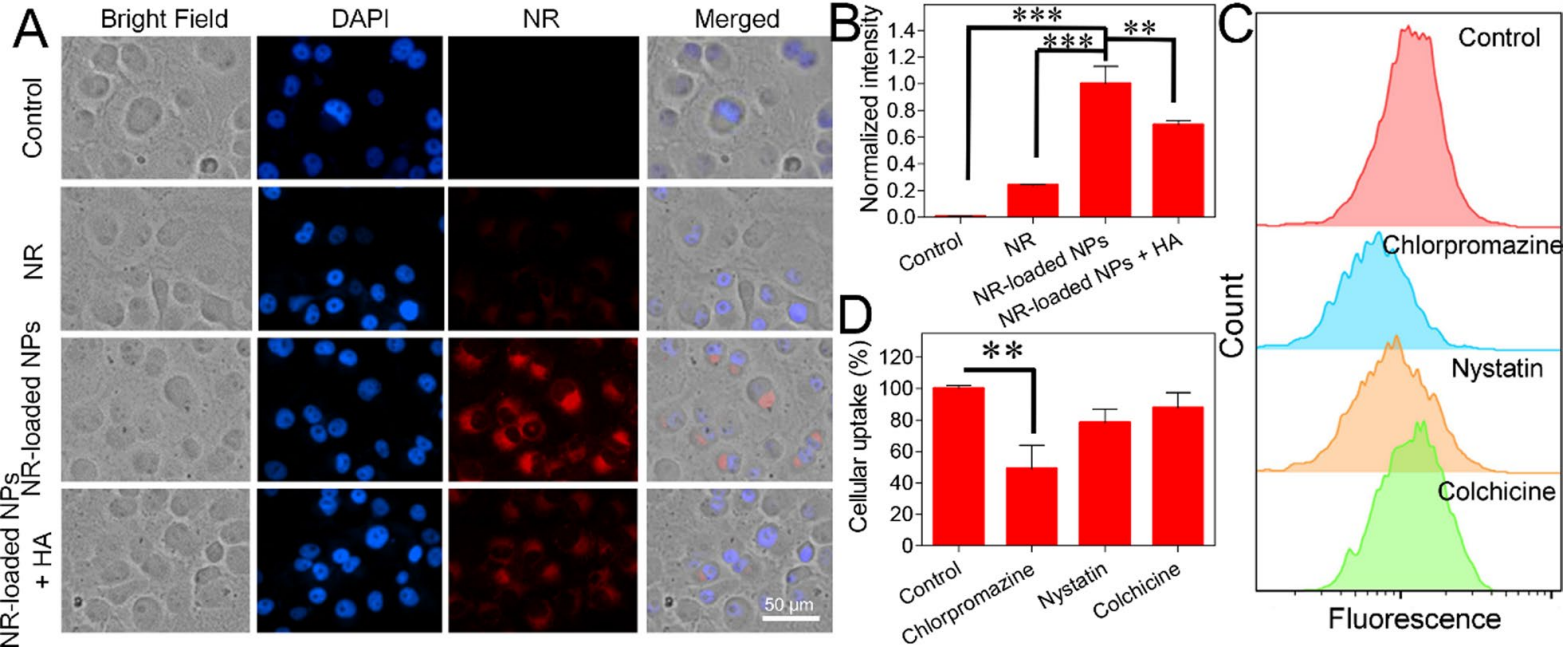
**A** Cellular uptake of free NR and NR-labeled PTFCG@MH measured by CLSM. **B** Intracellular fluorescence quantification for different groups. **C** Flow cytometric analysis of nanoparticles uptake in MDA-MB-231 cells with pre-treatment of various inhibitors. **D** The quantification of uptake from flow cytometric analysis. **P<0.01, ***P<0.001

We next tested the intracellular performance of the nanosystem by using MDA-MB-231 cancer cells. To track the intracellular delivery, nanoparticles were labeled with a red fluorescent Nile red (NR), and the cell nuclei were stained blue by DAPI for localization (Fig.3A). From confocal laser scanning microscopy (CLSM) images, a weak red signal was observed when the cells were treated with free NR, while the fluorescence was significantly intensified for nanoparticles. From the merged image, the fluorescence of nanoparticles mainly distributed in the cytoplasm, indicating endocytosis pathway for internalization. To demonstrate tumor targetability of such surface HA modified nanosystem, the cells were pretreated with free HA to saturate the CD44 binding, and in this case the intracellular nanoparticles signal decreased obviously. We also quantified the contribution of HA-mediated internalization by measuring the intensity of each treatment, where the uptake was significantly decreased upon free HA pre-treatment (Fig.3B). Using normal human umbilical vein endothelial cells (HUVEC) as control, the results further demonstrated the targetability of the nanoparticles towards tumor cells with significantly higher fluorescence (Additional file 1: Figure S6). These results confirmed that our nanosystem was able to selectively recognize tumor cells via CD44 receptor for targeting delivery. To further explore the delivery mechanism, various endocytosis inhibitors were employed, including chlorpromazine, nystatin and colchicine, which blocks clathrin-mediated endocytosis, caveolae-mediated endocytosis and micropinocytosis, respectively. Among then, chlorpromazine has the most significant impact on nanoparticles uptake (Fig.3C, D), indicating the main contribution of clathrin-mediated endocytosis.

### Enhanced anti-tumor efficacy via hypoxia alleviation and ATP/GSH depletion

**Fig. 4:**
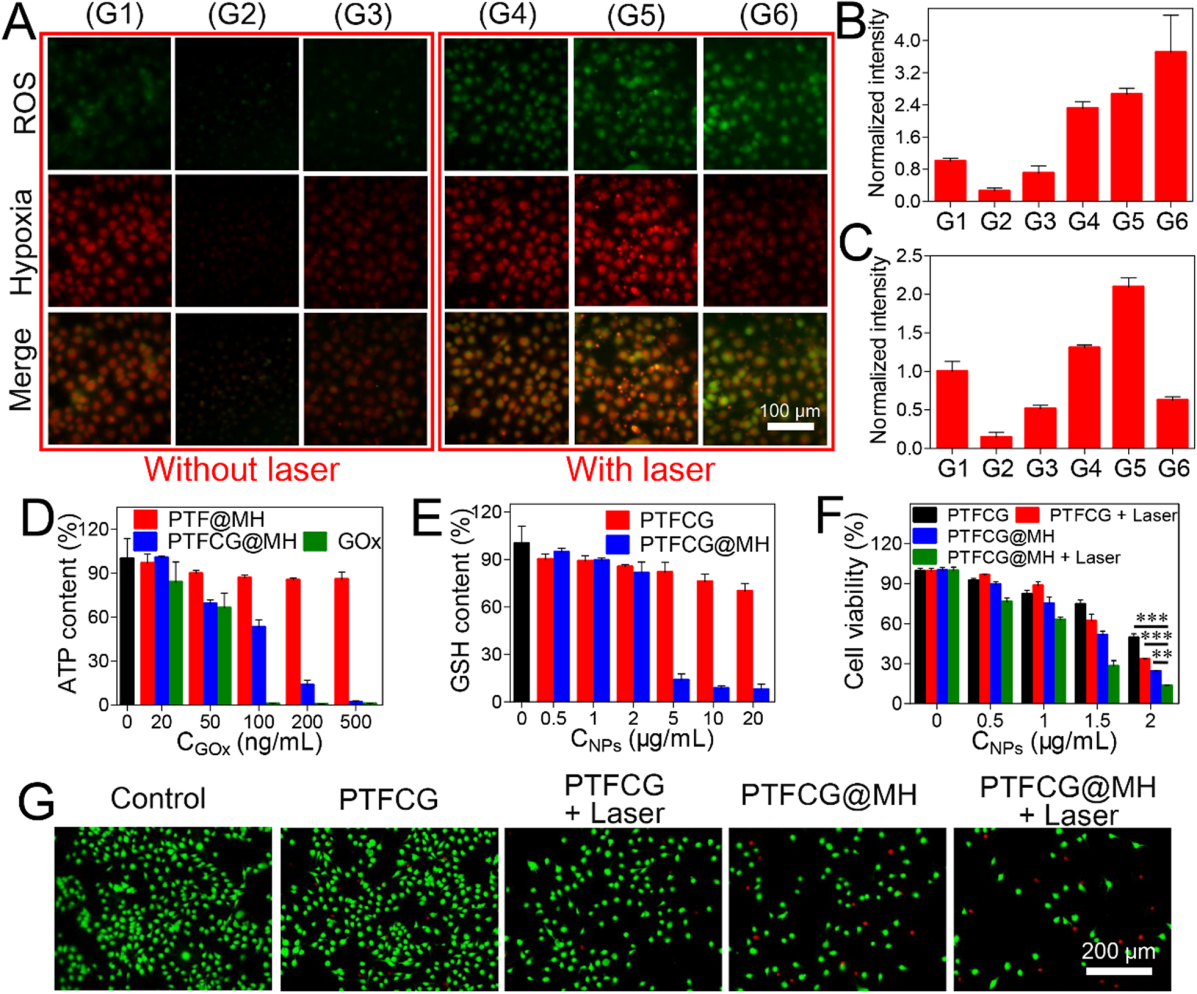
**A** Fluorescence images of intracellular ROS generation and hypoxia level with different treatments. The quantitative analysis of **B** ROS generation and **C** hypoxia level. In each panel, G1: Control; G2: PTF@MH; G3: PTFCG@MH; G4: Ce6+Laser; G5: PTFCG+Laser; G6: PTFCG@MH+Laser. The intracellular **D** ATP and **E** GSH content of MDA-MB-231 cells incubated with various formulations. **F** Concentration dependent cytotoxicity of different formulations for MDA-MB-231 cells. **G** Fluorescence images of cells with different treatments after co-staining with calcein-AM and propidium iodide. **P<0.01, ***P<0.001

After internalization, we next explored the intracellular functions of the nanosystem. To visualize the PDT effect, the ROS generation was probed by using 2 7'-dichlorofluorescin diacetate (DCFDA) indicator. Interestingly, both PTFCG@MH and PTF@MH could scavenge the intracellular ROS to some extent as compared to the control, due to the catalase activity of MnO_2_. Upon irradiation, each Ce6-based formulation showed enhanced green fluorescence inside cells based on both fluorescent images (Fig.4A) and quantified intensity (Fig.4B). Notably, PTFCG@MH group emitted the strongest fluorescence, indicating the best PDT efficacy. This can partially be explained by the cyclic oxygenation of the nano-reactor for self-oxygen supply. To confirm this, we then measured the O_2_ balance by using a red fluorescent hypoxia detection kit. Both PTF@MH and PTFCG@MH could relieve tumor hypoxia with red fluorescence decrease compared to non-treatment control (Fig.4A, C), also attributable to self-oxygen supply of the MnO_2_. Upon addition of laser, the hypoxia was strongly exacerbated for free Ce6 and PTFCG groups, ascribed to PDT- and GOx-based oxygen consumption. However, the PTFCG@MH group showed low hypoxia level even after laser irradiation. Therefore, such nano-reactor is robust enough to maintain oxygen balance with enhanced PDT efficacy.

Besides cyclic oxygen supply, such nano-reactor was also designed to deplete both ATP and GSH. We next tested these properties by measuring the intracellular ATP and GSH levels using ATP assay kit and Ellmans reagent, respectively. Because of catalytic glucose oxidation, free GOx could effectively decrease ATP level in a concentration dependent manner by blocking the energy supply (Fig.4D). Likewise, the PTFCG@MH could also inhibit ATP generation, while the PTF@MH did not show any activity due to the absence of GOx loading. MnO_2_ is a well-known GSH depletor due to its capability to oxidize GSH into GSSG, accompanied by its reduction into Mn^2+^ [28, 38]. As expected, the PTFCG@MH with MnO_2_ doping displayed high efficient GSH depletion, with over 80% GSH decrease at 5g/mL nanoparticles (Fig.4E). Note that ATP is the basic energy source for tumor cells to acquire treatments resistance including PDT therapy [39], and GSH could directly scavenge ^1^O_2_ to alleviate the PDT efficacy. Therefore, with ATP and GSH dual-depletion activity, such nano-reactor was expected to enhance the PDT-based anti-tumor activity via distinct mechanisms.

Next, the in vitro cytostatic activity of nanosystem was evaluated by MTT assay. Without drugs loading, the PTF and PTF@MH showed satisfactory biocompatibility even at high concentrations (Additional file 1: Figure S7). The PTFCG, on the other hand, displayed a concentration-dependent tumor ablation activity (Fig.4F), ascribed to the starvation therapy for ATP depletion [40]. Upon laser irradiation, the antitumor effect was further enhanced. Notably, PTFCG@MH showed significantly better efficacy than PTFCG attributable to MnO_2_ doping for self-oxygen circulation, GSH depletion, as well as HA modification for targeting delivery. To confirm the tumor selectivity, MTT assay was also performed on HUVEC cells, and as expected, higher cytotoxicity was observed for MDA-MB-231 cells (Additional file 1: Figure S8). We also explored the cell death pathway by co-staining the cells using calcein-AM (green fluorescence for live cells) and propidium iodide (red fluorescence for dead/late apoptotic cells). After different treatments, green fluorescence was weakened while the red fluorescence became intensified inside cells (Fig.4G), indicating an apoptotic or necrotic cell death mechanism. In addition, the general anti-tumor activity for each treatment was highly consistent with the results from MTT assay.

### In vivo performance of the nano-reactor

**Fig. 5:**
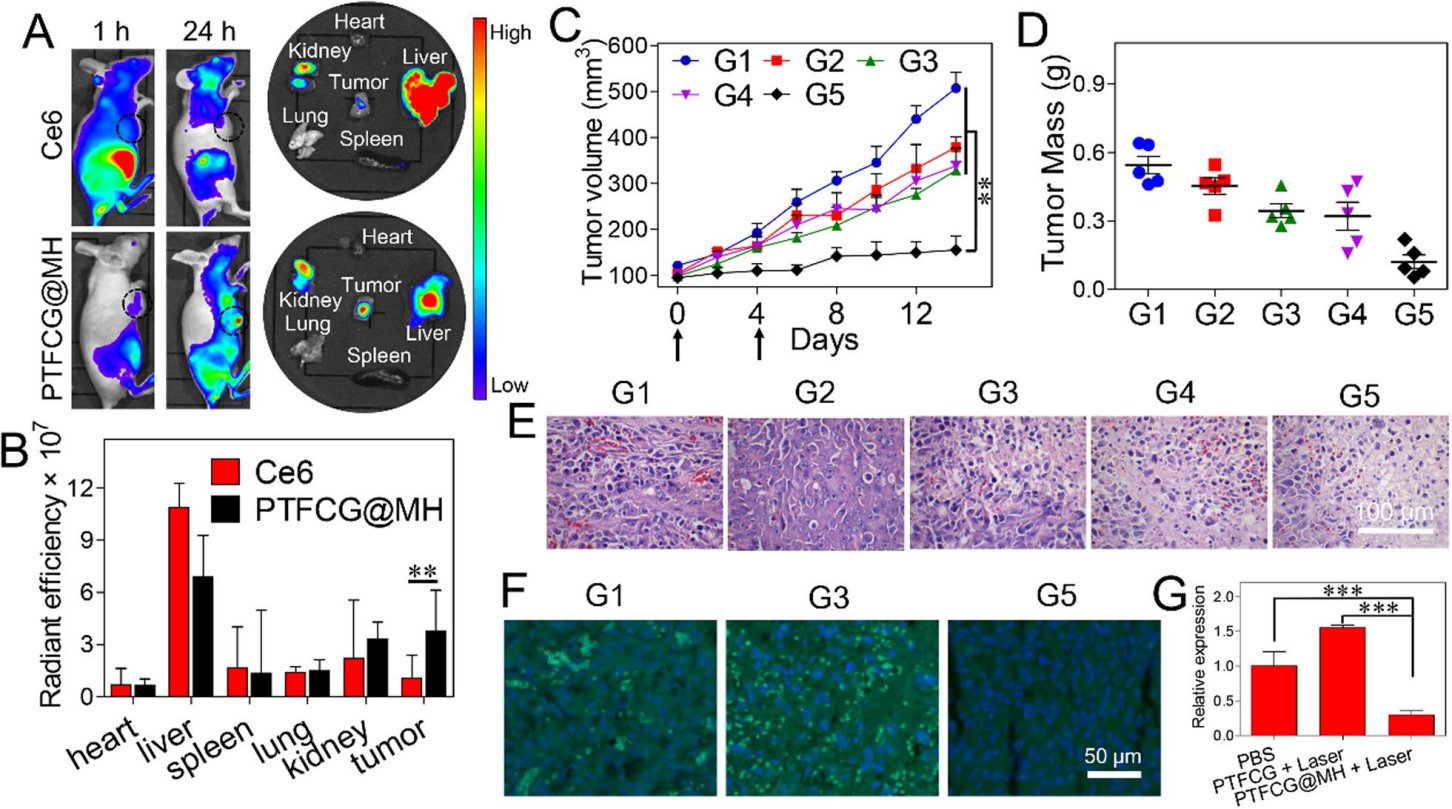
**A** In vivo (1h vs 24h post-injection) and ex vivo (24h post-injection) fluorescent imaging of MDA-MB-231 tumor-bearing mice treated with free Ce6 and PTFCG@MH. **B** The quantified fluorescence intensity in major organs and tumor tissue at 24h post injection. **C** Kinetics of tumor growth inhibition with various treatments. **D** Tumor mass and tumor images (inset) of the extracted tumor tissues after different treatments. **E** H&E staining and **F** HIF-1 immunofluorescence staining of the tumor tissues with different treatments. **G** The quantified HIF-1 level after different treatments. **P<0.01, ***P<0.001. In each panel, G1: PBS; G2: Ce6+Laser; G3: PTFCG+Laser; G4: PTFCG@MH; G5: PTFCG@MH+Laser

Finally, in vivo behavior of the nano-reactor was explored by using MDA-MB-231 tumor-bearing mice. By virtue of the intrinsic fluorescence of Ce6, the bio-distribution was visualized using a living imaging system. PTFCG@MH or free Ce6 was intravenously injected when the tumor volume reached~100mm^3^. At 1h post-injection, Ce6 showed red fluorescence throughout the body (Fig.5A), indicating non-specific distribution, while most of the fluorescence was cleared after 24h, with major accumulation in liver. For comparison, the PTFCG@MH displayed much weaker fluorescence at 1h post-injection due to the fluorescence quenching of the nanosystem, further demonstrating its advantage for decreased phototoxicity. However, the signal at tumor tissue became intensified after 24h circulation (Fig.5A, black circle), indicating the EPR effect of the nanomedicine for passive accumulation into tumor. We then quantified the results by collecting the main organs as well as tumor tissues for ex vivo fluorescence imaging (Fig.5B). PTFCG@MH exhibited~3.7-fold higher intensity at tumor site than free Ce6, confirming the targetability of the nanosystem towards tumor tissue. Meanwhile, we also noticed considerable accumulation of nanoparticles in liver and kidneys, the main organs to sequester and eliminate nanoparticles from body, and it was also observed in many other nanomedicines [41, 42]. Fortunately, temporal controlled activation of PDT would enable selective damage toward tumor, thus minimizing the unwanted side-effects.

To evaluate the in vivo tumor ablation activity, the tumor bearing mice were randomly divided into five groups (n=5), each intravenous injection of PBS, Ce6+Laser, PTFCG+Laser, PTFCG@MH, and PTFCG@MH+Laser, respectively (100L, [Ce6]=2.5mg/kg), with totally two injections (day 0 and day 4). The laser was performed at 24h after injection. The therapeutic efficacy was monitored by measuring the tumor size every other day (Fig.5C). Compared with PBS control, free Ce6 only showed marginal tumor growth inhibition, mainly due to its rapid clearance from mouse body with minimal accumulation into tumor tissue (Fig.5A). While the nanoparticles could facilitate tumor targeting delivery of Ce6, the efficacy of PTFCG plus laser irradiation was still poor, due to the multiple resistance mechanisms of tumor against PDT therapy. For PTFCG@MH, by contrast, the growth of tumors was obviously suppressed with a strong growth-inhibitory activity (~76%). Since we only performed two dosages and the treatment was stopped at day 4, a slight tumor recurrence was observed at day 6. However, the overall tumor growth was significantly suppressed for PTFCG@MH plus laser as compared with other treatment groups, demonstrating the superiority of the nanosystem for tumor therapy. Note that without laser irradiation, the therapeutic effect of PTFCG@MH was also rather limited, verifying that the anti-tumor activity was mainly originated from the PDT effect.

Next, the tumor tissues were extracted for a series of characterizations. Based on the tumor weight evaluations (Fig.5D), it was clearly seen that PTFCG@MH plus laser achieved the best efficacy, in consistent with the in vivo measurement. We also explored the pathological changes by H&E staining (Fig.5E), in which the tumor with PTFCG@MH plus laser treatment displayed widened interstitial space, nuclear condensation, and large vacuoles, indicating the highest level of tumor apoptosis. We also evaluated the key pathological feature of tumor hypoxia by immunofluorescence staining of HIF-1. The solid tumor showed a relatively high-level HIF-1 expression due to its hypoxia microenvironment (Fig.5F), and the immunofluorescence was even brighter after PTFCG treatment (with laser) because of the oxygen consumption by GOx catalysis and PDT. For comparison, the tumor hypoxia was effectively relieved after PTFCG@MH (plus laser) treatment, due to oxygenation by MnO_2_. From quantified results, the HIF-1 level was decreased~70% after PTFCG@MH treatment (Fig.5G). Therefore, the PTFCG@MH could effectively modulate tumor hypoxia microenvironment and provide O_2_ substrate to improve PDT treatment outcome.

Finally, the biosafety of the nanosystem was examined. No obvious decrease of body weight was observed over the period of treatments (Additional file 1: Figure S9), and the H&E staining showed no pathological change of the major organs after treatments (Additional file 1: Figure S10). These results indicate the biocompatibility of the nanosystem for in vivo applications.

## Conclusions

In conclusion, an intelligent cyclic nano-reactor, PTFCG@MH, was fabricated for enhanced PDT against solid tumor. The nanostructure was well-characterized, and the cyclic reactions were explicitly demonstrated in solution, including GOx-catalyzed glucose consumption, MnO_2_-mediated oxygenation and GSH depletion. The surface HA modification endowed the nano-reactor with improved colloidal stability and active targetability to facilitate its accumulation into tumor after intravenous injection. After being delivered into tumor cells, PTFCG@MH boosted the PDT efficacy via simultaneous ATP/GSH suppression and self-oxygen supply, resulting in efficient tumor growth inhibition with no obvious side-effects. This cascade nano-reactor would promote the development of multifunctional nanoplatforms for improved cancer treatment by modulating unfavorable cancer microenvironment.

## Supplementary Information

**Additional file 1.** Additional figures.

## Data Availability

All data generated or analysed during this study are included in this published article.

## Abbreviations

PDT: Photodynamic therapy
TME: Tumor microenvironment
GOx: Glucose oxidase
Ce6: Chlorin e6
PLGA: Poly (D, L-lactic-co-glycolic acid
MOF: Metalorganic framework
HA: Hyaluronic acid
GSH: Glutathione
PSs: Photosensitizers
ROS: Reactive oxygen species
HIF-1: Hypoxia inducible factor-1
HSPs: Heat shock proteins
P-gp: P-glycoprotein
ABCG2: ATP-binding cassette super-family G member 2
TA: Tannic acid
NR: Nile red
PAH: Poly (allylamine hydrochloride)
SOSG: Singlet oxygen sensor green
MTT: 3-(4, 5-Dimethylthiazol-2-yl)-2, 5-diphenyl tetrazolium bromide
DAPI: 4, 6-Diamidino-2-phenylindole
NAC: N-acetylcysteine
DCFDA: 2 7'-Dichlorofluorescin diacetate
EDS: Energy dispersive X-Ray spectroscopy
CLSM: Confocal laser scanning microscopy
