# Supplementary material for: A cyclic nano-reactor achieving enhanced photodynamic tumor therapy by reversing multiple resistances
Source: J Nanobiotechnology. 2021 May 21;19:149. doi: 10.1186/s12951-021-00893-6 (PMC8139056; doi:10.1186/s12951-021-00893-6)
Supplement: Supplementary file 1 — Additional file 1. Additional figures. [file 12951_2021_893_MOESM1_ESM.docx]

**Supporting Information**

**A Cyclic** **Nano-reactor Achieving Enhanced Photodynamic Tumor Therapy by Reversing Multiple Resistances**

Peng Liu^1^, Yanbin Zhou^1^, Xinyi Shi^1^, Yu Yuan^1^, Ying Peng^1^, Surong, Hua^3^, Qiange Luo^1^, Jinsong Ding^1^, Yong Li*^2^, Wenhu Zhou*^1^

^1^. Xiangya School of Pharmaceutical Sciences, Central South University, Changsha, Hunan, 410013, China

^2^. Department of Pediatric Surgery, Hunan Children’s Hospital, Changsha 410004, Hunan, China

^3^. Department of General Surgery, Peking Union Medical College Hospital, 100730, Beijing, China

*Email: [zhouwenhuyaoji@163.com](mailto:zhouwenhuyaoji@163.com)

**Figure S1.** The dynamic particle size of PTFCG, PTFCG@M and PTFCG@MH.

**Figure S2.** The TEM images of PTFCG@MH

**Figure S3.** The photographs of PTFCG@M and PTFCG@MH after incubation with PBS buffer (pH 7.4) for 24 h.

**Figure S4.** Fluorescence emission spectra of Ce6, PTFCG, PTFCG@MH and PTFCG@MH with GSH pretreatments. Inset: Fluorescence images of PTFCG@MH before and after pretreatments.

**Figure S5.** The Lineweaver-Burk plotting of PTFCG@MH + H_2_O_2_ (A) and PTFCG@MH (B).

**Figure S6.** (A) Cellular uptake of nanoparticles after incubating with HUVEC and MDA-MB-231 cells. (B) The quantitative analysis of cellular uptake. ** P < 0.01.

**Figure S7.** Cell viability of MDA-MB-231 cells treated with different concentrations of PTF and PTF@MH.

**Figure S8.** Concentration-dependent cytotoxicity of PTFCG@MH under laser irradiation after incubating with HUVEC and MDA-MB-231 cells. ** P < 0.01, *** P < 0.001.

**Figure S9.** Average body weights of mice with various treatments.

**Figure S10.** Histology images of H&E staining slices for major organs obtained from mice with different treatments.
